# Associations Among Religiosity, Religious Rejection, Mental Health, and Suicidal Ideation in Transgender and Gender Nonconforming Adults

**DOI:** 10.3390/bs15030270

**Published:** 2025-02-25

**Authors:** Steph L. Cull, Paul B. Perrin, Richard S. Henry

**Affiliations:** 1 Department of Psychology, Virginia Commonwealth University, Richmond, VA 23284, USA; culls@vcu.edu; 2 School of Data Science, University of Virginia, Charlottesville, VA 22903, USA; 3 Department of Psychology, University of Virginia, Charlottesville, VA 22903, USA; 4 Lady Davis Institute for Medical Research, Jewish General Hospital, Montreal, QC H3T 1E1, Canada; richard.henry@mail.mcgill.ca; 5 Department of Psychiatry, McGill University, Montreal, QC H3A 1A1, Canada

**Keywords:** transgender, non-binary, religiosity, discrimination, rejection

## Abstract

Objectives: Religiosity has generally been shown to be a protective factor against adverse mental health in the general population. Transgender and gender non-conforming (TGNC) individuals, however, may not experience the same protective effects, as many religions are unsupportive of diverse gender identities. This study examined whether increased religiosity and having been rejected by a religious community because of one’s gender identity were associated with mental health issues in TGNC individuals. Methods: A sample of TGNC adults (*n* = 154), predominantly from the United States, completed an online survey assessing these constructs. Results: These constructs were highly represented in the sample, with 46.1% of participants having experienced religious rejection at some point throughout their lifetime because of their gender identity, 40.3% currently experiencing symptoms of elevated depression and 34.4% of elevated anxiety, and 48.7% with suicidal ideation over the past 2 weeks. Religious rejection was associated with increased depression, anxiety, and suicidal ideation. Neither interpersonal nor intrapersonal religiosity was associated in a bivariate way with any of the three mental health outcomes. However, among participants who experienced rejection by one’s religious community, interpersonal religiosity was strongly associated with increased depression symptoms and suicidal ideation, whereas conversely among TGNC individuals who had not experienced rejection by their religious community, interpersonal religiosity was weakly associated with depression and suicidal ideation. Conclusion: The results underscore the extremely harmful effects of religious rejection due to one’s gender identity on religious TGNC individuals, pointing to the active contributions of the behaviors of traditional religious groups towards TGNC mental health problems and suicide.

## 1. Introduction

Religiosity refers to an individual’s adherence to beliefs, worldviews, and practices of religion (Worthington et al., 2003). Religiosity is commonly viewed through two lenses: interpersonal (e.g., connection to a religious community, attending religious services) and intrapersonal (e.g., personal connection to religion, private prayer). Religiosity can be a protective factor against adverse mental health outcomes, including depression, anxiety, and suicidality among the general population (Giannone et al., 2019). Lytle et al. (2015) found that greater importance of religion in one’s life was protective against both suicidal ideation and attempts among heterosexuals, but not among sexual minorities. This suggests the same protective effects of religiosity cannot be directly extrapolated to the LGBTQIA+ (lesbian, gay, bisexual, transgender, queer, intersex, asexual, and other sexual and gender minorities) population. Research, however, has developed many nuances, with religion being both stressful and supportive for LGBTQIA+ individuals (Barnes & Meyer, 2012; Kralovec et al., 2014; Szymanski & Carretta, 2019).

When compared to heterosexual and cisgender populations, LGBTQIA+ individuals have higher rates of suicidal ideation and suicide attempts (Haas et al., 2010; Lytle et al., 2014). Members of minoritized groups experience unique stressors such as discrimination and stigmatization due to their marginalized identities (Hatzenbuehler & Pachankis, 2016; Meyer, 2003). For sexual minority individuals who actively engage in religion, minority stress may be even greater; Lefevor et al. (2022) found that minority stress processes such as internalized homonegativity and concealment were particularly prominent among sexual minority members of a conservative religious tradition (LDS). Another study found that sexual and gender minorities who identified as Christian, either in childhood or adulthood, were more likely to experience stress and stigma (Lefevor et al., 2023a). Research has found between 1.5 and 5 times higher rates of mental health disorders and suicidality among sexual minorities as compared to the general population (Graugaard et al., 2015).

In general, individuals with traditional religious affiliations have negative attitudes towards LGBTQIA+ identities (Rowatt et al., 2009). Likewise, associations have been found between internalized heterosexism and religious discrimination, especially in the context of non-affirming religions (Barnes & Meyer, 2012; Kralovec et al., 2014). Extant research has shown that religiosity can serve as a protective factor for LGB individuals who have lower levels of religiosity but can exacerbate adverse outcomes such as internalized stigma for those who have high religiosity (Szymanski & Carretta, 2019). There is a plethora of evidence illustrating the ostracism/rejection that sexual minority individuals face from more traditional religious institutions (Lytle et al., 2013), despite some affirming religious organizations, such as Unitarian Universalism, Episcopalian Christianity, and Reform Judaism. Although a small body of research has examined associations among religious experiences and mental health among the broader LGBTQIA+ community, and in particular sexual minorities (Barnes & Meyer, 2012; Kralovec et al., 2014; Lefevor et al., 2022, 2023b; Lytle et al., 2015; Szymanski & Carretta, 2019), very little research has focused on religiosity and religious experiences within the transgender/gender non-confirming (TGNC) community. Many studies examining mental health outcomes within the LGBTQIA+ population have extremely small TGNC subsamples, sometimes less than 1%.

As a result of this combination of gaps in the literature, the purpose of the current study was to examine associations among religiosity, religious rejection due to one’s gender identity, mental health, and suicidal ideation in TGNC adults. It was hypothesized that experiences with religious rejection would amplify (or moderate) the effect of religiosity on mental health and suicidal ideation, such that religiosity in TGNC individuals would only lead to adverse mental health outcomes when TGNC individuals have experienced rejection by their religious community because of their gender identity. We hypothesized that religiosity would be related to poorer mental health outcomes specifically for individuals who had been rejected by their religious community due to their gender identity, consistent with the findings of Szymanski and Carretta (2019) that religious-based sexual stigma in LGB individuals predicted increased psychological distress at high, but not low, levels of religiosity; as a result, in the current study, we expected that religious rejection would exacerbate the link between religiosity and mental health outcomes in TGNC individuals.

## 2. Materials and Methods

### 2.1. Participants

Participants included individuals who currently or have ever self-identified as TGNC (*n* = 154). The vast majority of participants were from the United States (*n* = 152), and the average age was 29.89 (*SD* = 8.2). See Table 1 for additional demographic information.

### 2.2. Procedure

Data for this study were part of a larger survey on the health needs and discrimination experiences of TGNC individuals (Henry et al., 2020). The survey for this study was hosted online via Qualtrics and took participants approximately an hour to complete. Data were collected via two online mechanisms. First, snowball sampling was used involving a TGNC listserv of individuals who had previously participated in research (Witten, 2014). Two recruitment emails were sent, approximately two weeks apart, using the listserv. The emails contained a description of the study, informed consent form, a link to the study screener, and the survey. Second, the study utilized Amazon’s Mechanical Turk (MTurk). MTurk is a popular data crowdsourcing platform that deploys surveys (called human intelligence tasks or HITs) and compensates the participants (called workers) with small monetary amounts. Participants in this study were compensated with $1 USD, an amount which is consistent with previous MTurk research studies. Since MTurk does not allow for the collection of identifying information, all surveys completed through this platform were anonymous. Using these two online techniques, 154 participants were recruited: seven from the listserv and 147 from MTurk.

Inclusion criteria for this study were that participants must: (a) currently identify or have previously identified as transgender, gender non-conforming, or gender non-binary, (b) be able to complete the entire survey in English, (c) be at least 18 years of age, and (d) have access to the internet or a mobile device that would allow them to access and complete the survey. The study was approved by the host university’s institutional review board, and all participants provided informed consent.

### 2.3. Measures

Depressive Symptoms. Depressive symptoms were measured using the first eight items of the Patient Health Questionnaire-9 (PHQ-9; Kroenke et al., 2001). The PHQ-9 is a nine-item scale with responses that range from 0–3, for a total possible score of 0–27. The items measure symptomology over the previous two-week period, and higher scores reflect higher depressive symptomology. A final question on the PHQ-9 asks participants, “[Over the past two weeks, how often have you been bothered by] thoughts that you would be better off dead or hurting yourself in some way?” which was used separately to assess suicidality.

Anxiety Symptoms. Anxiety symptoms were measured with the Generalized Anxiety Disorder-7 (GAD-7; Spitzer et al., 2006). The GAD-7 is a seven-item measure with responses ranging from 0–3, where higher summed scores reflect higher levels of anxiety symptoms in the previous two weeks.

Religiosity. For this survey, religiosity was assessed using the Religious Commitment Inventory-10 (RCI-10; Worthington et al., 2003). The RCI-10 is a ten-item measure that assesses levels of both interpersonal religiosity (e.g., religious community, attending religious services, connections with a religious congregation) and intrapersonal religiosity (e.g., an individual’s personal connection with religion or spirituality, prayer, reading religious texts) where higher scores indicate higher levels of religious commitment, or religiosity. For this study, we used sum scores representing intrapersonal religiosity (6 questions) and interpersonal religiosity (4 questions; α = 0.94). The RCI-10 has answer options of “not at all true of me, somewhat true of me, moderately true of me, mostly true of me, totally true of me”.

Religious Rejection. One item from the Gender Minority Stress and Resilience Scale (GMSR) was used to assess religious rejection in this sample: “I have been rejected or made to feel unwelcome by a religious community because of my gender identity or expression”. Participants respond to this item with four options of “never, yes before age 18, yes after age 18, or yes in the past year”. Participants who responded “never” were coded as a 0, indicating that they had not experienced religious rejection, and participants who chose any of the other 3 response options were coded as a 1, indicating that they had experienced religious rejection at some point during their lifetime.

### 2.4. Statistical Analyses

Six hierarchical regressions were performed in which the predictor variable was intrapersonal or interpersonal religiosity, the moderator was religious rejection, and the dependent variable was depression, suicidal ideation, or anxiety. Each model had three steps with (a) one of the forms of religiosity as the predictor, (b) religious rejection added as an additional predictor, and (c) the religiosity*religious rejection as a third predictor. All predictors were mean-centered. Given that no data were missing in the current study, no imputation techniques were used.

## 3. Results

### 3.1. Preliminary Analyses

Preliminary analyses illustrating the descriptive statistics and bivariate correlations of the measures of interest are summarized in Table 2 and Table 3. In this sample, 46.1% of participants had experienced religious rejection at some point throughout their lifetime, 40.3% had elevated depressive symptoms and 34.4% elevated anxiety symptoms within the last 2 weeks, and 48.7% had experienced suicidal ideation over the past 2 weeks. In the correlation matrix (Table 3), religious rejection was positively associated with suicidal ideation, depression, and anxiety, but not either form of religiosity. Suicidal ideation, depression, and anxiety were all positively associated with each other.

### 3.2. Regression 1: Intrapersonal Religiosity and Depression

Block 1 with intrapersonal religiosity as the sole predictor of depression was not significant, *F*(1, 152) = 2.20, *p* = 0.140, *R*^2^ = 0.01. Block 2 with religious rejection as the second predictor improved the model significantly, Δ*R*^2^ = 0.05, *p* = 0.005. Block 3 with the intrapersonal religiosity*religious rejection addition did not improve the model, Δ*R*^2^ = 0.01, *p* = 0.131. In this final block, religious rejection was the only predictor uniquely associated with increased depression, β = 0.23, *p* = 0.005. The interaction between religious rejection and intrapersonal religiosity was not significant, β = 0.12, *p* = 0.131, indicating no moderating effect of religious rejection on the relationship between intrapersonal religiosity and depression.

### 3.3. Regression 2: Interpersonal Religiosity and Depression

**Figure 1:**
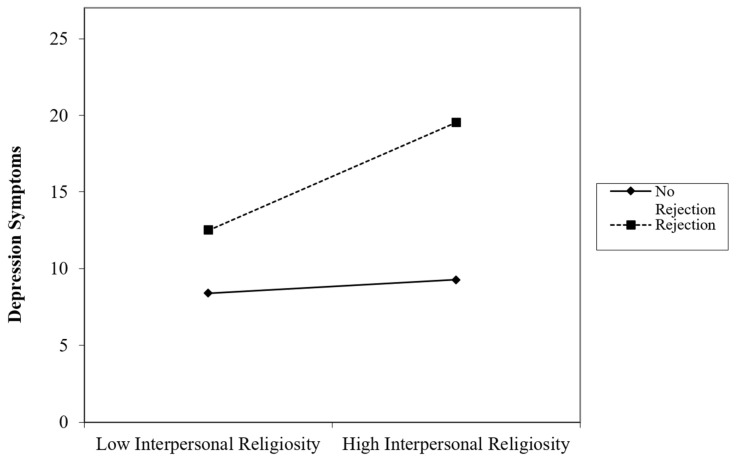
Interpersonal Religiosity and Religious Rejection Interaction on Depression.

Block 1 with interpersonal religiosity as the sole predictor of depression was not significant, *F*(1, 152) = 1.25, *p* = 0.265, *R*^2^ = 0.01. Block 2 with religious rejection as the second predictor improved the model significantly, Δ*R*^2^ = 0.05, *p* = 0.004. Block 3 with the interpersonal religiosity*religious rejection addition improved the model significantly, Δ*R*^2^ = 0.02, *p* = 0.048. In this final block, religious rejection was again uniquely associated with increased depression, β = 0.23, *p* = 0.004. The interaction between religious rejection and interpersonal religiosity was also significant, β = 0.16, *p* = 0.048, indicating a moderating effect of religious rejection on the relationship between interpersonal religiosity and depression (Figure 1). For TGNC individuals who had not experienced religious rejection, interpersonal religiosity was weakly associated with depression. For TGNC individuals who had experienced religious rejection, interpersonal religiosity was strongly and positively associated with depression.

### 3.4. Regression 3: Intrapersonal Religiosity and Suicidal Ideation

Block 1 with intrapersonal religiosity as the sole predictor of suicidal ideation was not significant *F*(1, 152) = 1.31, *p* = 0.255, *R*^2^ = 0.01. Block 2 with religious rejection as the second predictor improved the model significantly, Δ*R*^2^ = 0.03, *p* = 0.043. Block 3 with the intrapersonal religiosity*religious rejection addition did not improve the model, Δ*R*^2^ = 0.01, *p* = 0.201. The interaction between religious rejection and intrapersonal religiosity was not significant, β = 0.10, *p* = 0.201, indicating no moderation of religious rejection on the relationship between intrapersonal religiosity and suicidal ideation.

### 3.5. Regression 4: Interpersonal Religiosity and Suicidal Ideation

**Figure 2:**
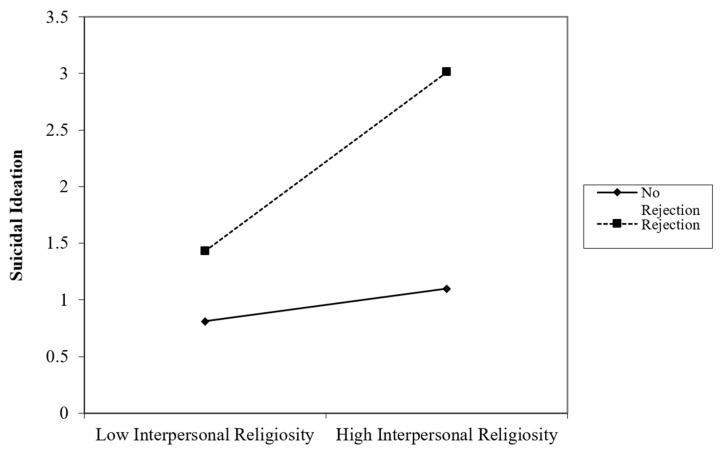
Interpersonal Religiosity and Religious Rejection Interaction on Suicidal Ideation.

Block 1 with interpersonal religiosity as the sole predictor of suicidal ideation was not significant, *F*(1, 152) = 2.37, *p* = 0.125, *R*^2^ = 0.02. Block 2 with religious rejection as the second predictor improved the model significantly, Δ*R*^2^ = 0.025, *p* = 0.048. Block 3 with the interpersonal religiosity*religious rejection addition improved the model even further, Δ*R*^2^ = 0.04, *p* = 0.013. In this block, religious rejection was significant, β = 0.16, *p* = 0.048, and the interaction between religious rejection and interpersonal religiosity was also significant, β = 0.20, *p* = 0.013, indicating a moderating effect of religious rejection on the relationship between interpersonal religiosity and suicidal ideation (Figure 2). For TGNC individuals who had not experienced religious rejection, interpersonal religiosity was weakly associated with suicidal ideation. For TGNC individuals who had experienced religious rejection, interpersonal religiosity was strongly and positively associated with suicidal ideation.

### 3.6. Regression 5: Intrapersonal Religiosity and Anxiety

Block 1 with intrapersonal religiosity as the sole predictor of anxiety was not significant, *F*(1, 152) = 0.005, *p* = 0.943, *R*^2^ = 0.00. Block 2 with religious rejection as the second predictor improved the model significantly, Δ*R*^2^ = 0.08, *p* < 0.001. Block 3 with the intrapersonal religiosity*religious rejection addition did not improve the model, Δ*R*^2^ = 0.00, *p* = 0.873, indicating no significant moderating effect of religious rejection on the relationship between intrapersonal religiosity and anxiety. In this block, religious rejection was significant, β = 0.29, *p* < 0.001.

### 3.7. Regression 6: Interpersonal Religiosity and Anxiety

Block 1 with interpersonal religiosity as the sole predictor of anxiety was not significant *F*(1, 152) = 0.04, *p* = 0.848, *R*^2^ = 0.01. Block 2 with religious rejection as the second predictor improved the model significantly, Δ*R*^2^ = 0.08, *p* < 0.001. Block 3 with the interpersonal religiosity*religious rejection addition did not improve the model, Δ*R*^2^ = 0.005, *p* = 0.382, indicating no moderating effect of religious rejection on the relationship between interpersonal religiosity and anxiety. In this model, religious rejection was significant, β = 0.29, *p* < 0.001. Full regression results appear in Table 4.

## 4. Discussion

This study examined whether increased religiosity and having been rejected by a religious community because of one’s gender identity were associated with increased mental health issues in TGNC individuals. It was hypothesized that religious rejection would have an exacerbating effect on the relationship between religiosity and mental health. These constructs were highly represented in the current sample, with 46.1% of participants having experienced religious rejection at some point throughout their lifetime because of their gender identity, 40.3% currently experiencing symptoms of elevated depression and 34.4% of elevated anxiety, and 48.7% with suicidal ideation over the past 2 weeks. Religious rejection was associated with increased depression, anxiety, and suicidal ideation. Neither interpersonal nor intrapersonal religiosity was associated in a bivariate way with any of the three mental health outcomes. However, among participants who had experienced rejection by one’s religious community, interpersonal religiosity was positively and strongly associated with increased depression symptoms and suicidal ideation, whereas, conversely, among TGNC individuals who had not experienced rejection by their religious community, interpersonal religiosity was weakly associated with depression symptoms and suicidal ideation.

### 4.1. Mental Health Issues and Religious Rejection

This study’s finding of extremely high rates of anxiety and depression in TGNC adults is consistent with previous research finding higher rates of anxiety, depression, suicidal ideation, and substance use in the LGBTQIA+ population compared to the general population (Graugaard et al., 2015; Haas et al., 2010; Lytle et al., 2014, 2015). It is likely that these mental health issues in the current sample channeled directly into the extremely high rate of suicidal ideation, with nearly half of participants experiencing suicidal ideation over the last two weeks. This figure is consistent, though slightly higher, than that found in previous research, wherein rates of suicidal ideation and attempts in gender minority populations have been shown to be around 40% (James et al., 2016).

The high rates of religious rejection in the current sample (46.1%) were directly associated with depression, anxiety, and suicidal ideation. This is consistent with previous research showing religious rejection may exacerbate adverse mental health outcomes in the LGBTQIA+ community (Barnes & Meyer, 2012; Kralovec et al., 2014; Lytle et al., 2015). Many traditional and/or non-affirming religious groups have negative attitudes towards LGBTQIA+ identities (Barnes & Meyer, 2012; Kralovec et al., 2014; Rowatt et al., 2009); these attitudes have been shown to lead to rejection, stigmatization, and ostracism of members of the LGBTQIA+ community (Lytle et al., 2013) and to be related to increased minority stress. Given that experiences of stigmatization and discrimination have been associated with increased adverse mental health outcomes (Hatzenbuehler & Pachankis, 2016; Meyer, 2003) and that rates of adverse mental health problems are elevated in the LGBTQIA+ population (Haas et al., 2010; Lytle et al., 2014, 2015), the current findings are consistent with this body of research. These findings also expand on findings that religiosity can exacerbate adverse outcomes such as internalized stigma for those who have high religiosity (Szymanski & Carretta, 2019).

### 4.2. Moderating Role of Religious Rejection

Neither interpersonal nor intrapersonal religiosity was associated in a bivariate way with any mental health outcome. These findings are unlike results in the general population that found religiosity is a protective factor against adverse mental health outcomes, including depression, anxiety, suicidal ideation, and substance use (Giannone et al., 2019; Lytle et al., 2015). Taken together, these findings suggest that the same generally salubrious effects of religiosity likely do not occur in the same way in the TGNC community as for the general (cisgender, heterosexual) population.

When religious communities are supportive of TGNC individuals, the current study’s findings suggest that interpersonal religiosity may weakly, or not, be associated with depression and suicidal ideation, possibly serving as a buffering effect in a similar way as for heterosexual cisgender individuals (Giannone et al., 2019; Lytle et al., 2015). However, in non-supportive and rejecting religious communities, interpersonal religiosity likely increases risk for depression and suicidal ideation in TGNC individuals, findings which are similar to work that was conducted on sexual minority populations illustrating links between religiosity and minority stress processes (Lefevor et al., 2022, 2023b). For those who value connection to a religious community, religious rejection may be much more difficult to handle, leading to adverse mental health outcomes. Given that many of the mainstream and traditional branches of predominant religions in the United States (Christianity, Islam, Judaism) are generally non-affirming of LGBTQIA+ identities (Rowatt et al., 2009), these findings are incredibly pertinent, especially amidst the backdrop of high rates of mental health problems in the broader LGBTQIA+ community and particularly among TGNC individuals (Haas et al., 2010; Lytle et al., 2014, 2015).

Despite this moderating effect of interpersonal religiosity, intrapersonal religiosity was not associated with any mental health outcome, even among participants who had been rejected by their religious community for their gender identity. These findings make sense when considering that intrapersonal religiosity has more to do with one’s personal relationship with a higher power and/or spirituality, whereas interpersonal religiosity pertains more to one’s relationship with a religious community. Therefore, those TGNC individuals who have stronger personal relationships with spirituality (intrapersonal religiosity) may not be as adversely affected by religious (institutional) rejection. Those TGNC individuals who have stronger intrapersonal religiosity may experience the buffering effects that religiosity can have on adverse mental health outcomes as they are not as reliant on the communal aspects of interpersonal religiosity. Therefore, aiding TGNC individuals in cultivating their intrapersonal religiosity may lead to beneficial mental health outcomes.

### 4.3. Implications

Religious community leaders should be made aware of the dire impact of their and their congregation’s lack of support for TGNC populations. As religiosity tends to be a protective factor against adverse mental health outcomes in the general population (Giannone et al., 2019; Lytle et al., 2015), and LGBTQIA+ populations experience increased rates of adverse mental health outcomes (Haas et al., 2010; Lytle et al., 2014; Lytle et al., 2015), it is essential that religious leaders work to combat not only the overt negativity that many LGBTQIA+ individuals face in traditional religious settings but also initiate conversations about the actual theology condemning TGNC identities from otherwise seemingly accepting religious communities and denominations. Religious rejection can of course be overt, but it can also be covert when religious communities use language of the TGNC community, are nice to TGNC individuals, and welcome them into their congregation, but nonetheless “love the sinner and hate the sin”. Taking a critical look at theology is vital if it is dissonant from empirical evidence—like that in the current study—suggesting that rejection directly stemming from it increases depression and suicidal ideation in TGNC individuals who are otherwise religious and value and desire a healthy interpersonal connection to their religious community. There are numerous online resources available to religious leaders who desire to better support the LGBTQIA+ community, including the Training for Trainers to Help Build Inclusive Churches, offered by the National LGBTQ Task Force (https://www.thetaskforce.org/first-ever-training-for-trainers-to-help-build-inclusive-churches/; National LGBTQ Task Force, 2012; accessed on 7 January 2025), and the Reconcile and Reform Conference, offered by The Reformation Project (https://reformationproject.org/conference-2025/; The Reformation Project, 2023; accessed on 7 January 2025). There are also websites that offer resources including the LGBT Resources page of the United Church of Christ website (https://www.ucc.org/what-we-do/justice-local-church-ministries/justice/health-and-wholeness-advocacy-ministries/lgbtq-ministries/lgbt_resources/; United Church of Christ, 2022; accessed on 7 January 2025), the Believe Out Loud website (https://www.believeoutloud.com; Cathedral of Hope United Church of Christ, 2021; accessed on 7 January 2025), the Advancing LGBTQ Inclusion in the Church page of the Reformation Project (https://reformationproject.org/home-pride/; The Reformation Project, 2023; accessed on 7 January 2025), and the Faith-Based Resources on the LGBTQ Family Acceptance website (https://lgbtqfamilyacceptance.org/faith-based-resources/; Innovations Institute, 2023; accessed on 7 January 2025). By exploring these resources and actively learning how to become an ally to members of the LGBTQIA+ community, religious leaders can help combat the adverse mental health outcomes experienced by some religious members of the LGBTQIA+ community.

### 4.4. Limitations and Future Directions

The current study has several limitations, and as a result, directions for future research. Participants’ religious affiliation in the current sample generally was comprised of atheism and Christianity, with very small representation of TGNC individuals from other religious traditions. Therefore, no comparisons could be made among individuals of different religious traditions, and particularly among those that are affirming of diverse TGNC identities. Future research would benefit from recruiting TGNC individuals identifying with other major religious traditions. Further, the index of religious rejection was a single-item measure rather than a more thorough, psychometrically valid multi-item scale. As a result, no nuanced information was collected regarding circumstances surrounding religious rejection, and it is unknown whether participants had experienced overt, covert, violent, or other forms of rejection. Future research should explore the nuanced role of religiosity and religious rejection by incorporating a more robust and validated assessment of religious rejection. This would provide greater detail about rejection to help determine what specific types of religious rejection might be the most damaging.

## 5. Conclusions

This study found that religious rejection has a moderating effect on the relationships between interpersonal religiosity and both depression and suicidal ideation within a TGNC sample. As the body of research on the relationship between mental health and religiosity in gender diverse populations is limited, these findings add to extant research exploring these associations within the broader LGBTQIA+ population. The results underscore the extremely harmful effects of religious rejection due to one’s gender identity on religious TGNC individuals, pointing to traditional religious groups behaviors’ active contributions to TGNC mental health problems and suicide.

## Figures and Tables

**Table 1:** Demographics.

Variable	*n*	%
**Sexual Orientation**		
Straight/heterosexual	18	11.7
Bisexual	49	31.8
Gay	21	13.6
Lesbian	11	7.1
Queer	15	9.7
Pansexual	22	14.3
Asexual	17	11.0
Other	1	0.6
**Race/Ethnicity**		
White/European-American	104	67.5
Black/African-American	17	11.0
Asian/Asian-American/Pacific Islander	13	8.4
Latino/Hispanic	13	8.4
American Indian/Native-American/Alaska-Native	2	1.3
Multiracial/Multiethnic	5	3.2
**Household Income**		
<$10,000	8	5.2
Between $10,000 and $24,999	30	19.5
Between $25,000 and $39,999	27	17.5
Between $40,000 and $54,999	36	23.4
Between $55,000 and $69,999	24	15.6
Between $70,000 and $79,999	15	9.7
>$80,000	14	9.1
**Sex assigned at birth**		
Male	93	60.8
Female	58	37.9
Intersex	1	0.7
Disorders of sexual development	1	0.7
**Gender Self-Perception**		
Transmasculine	18	11.7
Masculine	23	14.9
Transfeminine	19	12.3
Feminine	56	36.4
Genderqueer/Gender non-conforming	36	23.4
Other	2	1.3
**Education**		
Doctorate Degree	1	0.6
Master’s Degree	17	11.0
4-Year College Degree	56	36.4
2-Year College Degree	26	16.9
Some College/No Degree	38	24.7
High School/GED	15	9.7
Grade School	1	0.6
**Religion**		
Atheist	82	53.2
Christian	38	41.2
Buddhist/Confucian	5	3.2
Muslim/Islam	5	3.2
Paganist/New Age Religions	3	1.9
Jewish	5	3.2
Other	1	0.6
None	5	3.2

Note. Bolded words reflect the overall demographic category.

**Table 2:** Descriptive statistics.

Variable	Min	Max	95% CI	Mean	*SD*	Skew	Kurt.	α
Anxiety	0.00	21.00	6.51, 8.20	7.36	5.32	0.41	−0.60	0.91
Depression	0.00	25.00	8.10, 10.07	9.08	6.18	0.23	−0.70	0.90
Suicidality	0	3	0.62, 0.91	0.77	0.93	0.97	−0.12	-
Intrapersonal religiosity	6.00	30.00	11.60, 13.80	12.70	6.91	0.61	−0.80	0.93
Interpersonal religiosity	4.00	20.00	7.50, 8.94	8.21	4.60	0.70	−0.58	0.94
Religious rejection	0.00	1.00	0.38, 0.54	0.46	0.50	0.16	−2.00	-

**Table 3:** Bivariate correlations.

Variable	1	2	3	4	5
1. Suicidality	-				
2. Depression	0.58 **				
3. Anxiety	0.50 **	0.78 **			
4. Religious Rejection	0.18 *	0.24 **	0.28 **		
5. Intrapersonal Religiosity	0.09	0.12	−0.01	0.17 *	
6. Interpersonal Religiosity	0.12	0.10	−0.03	0.15	0.91 **

**Note.** ** = *p* < 0.001; * = *p* < 0.05.

**Table 4:** Regression summary.

Model	Predictor	Depression		Suicidal Ideation		Anxiety	
		β	*p*	β	*p*	β	*p*
1	Intrapersonal Religiosity	0.12	0.140	0.09	0.255	−0.01	0.943
2	Intrapersonal Religiosity	0.08	0.310	0.06	0.428	−0.06	0.485
	Religious Rejection	0.23	0.005	0.17	0.043	0.29	<0.001
3	Intrapersonal Religiosity	0.06	0.429	0.05	0.548	−0.05	0.506
	Religious Rejection	0.23	0.005	0.17	0.043	0.29	<0.001
	Intrapersonal Religiosity * Religious Rejection	0.12	0.131	0.10	0.201	−0.01	0.873
		Depression		Suicidal Ideation		Anxiety	
Model	Predictor	β	*p*	β	*p*	β	*p*
1	Interpersonal Religiosity	0.09	0.265	0.12	0.125	−0.02	0.848
2	Interpersonal Religiosity	0.06	0.493	0.10	0.221	−0.06	0.441
	Religious Rejection	0.23	0.004	0.16	0.048	0.29	<0.001
3	Interpersonal Religiosity	0.05	0.568	0.09	0.274	−0.07	0.412
	Religious Rejection	0.23	0.004	0.16	0.048	0.29	<0.001
	Interpersonal Religiosity * Religious Rejection	0.16	0.048	0.20	0.013	0.07	0.382

Note. * = interaction term.

## Data Availability

Data is available from the corresponding author upon request.
